# The clinical benefit of cardiac resynchronization therapy optimization using a device-based hemodynamic sensor in a patient with dilated cardiomyopathy: a case report

**DOI:** 10.1186/s13256-015-0761-y

**Published:** 2015-12-19

**Authors:** Mario Volpicelli, Gregorio Covino, Paolo Capogrosso

**Affiliations:** H. San Giovanni Bosco, Naples, Italy

**Keywords:** Automatic CRT optimization, Cardiac resynchronization therapy (CRT), Responder to CRT

## Abstract

**Introduction:**

Results on the evolution of the clinical status of patients undergoing cardiac resynchronization therapy with a defibrillator after automatic optimization of their cardiac resynchronization therapy are scarce. We observed a rapid and important change in the clinical status of our non-responding patient following activation of a sensor capable of weekly atrioventricular and interventricular delays' optimization.

**Case presentation:**

A 78-year-old Caucasian man presented with dilated cardiomyopathy, left bundle branch block, a left ventricular ejection fraction of 35 %, New York Heart Association class III/IV heart failure, and paroxysmal atrial fibrillation. Our patient was implanted with a cardiac resynchronization device with a defibrillator and the SonRtip atrial lead. Right ventricular and left ventricular leads were also implanted. Because of the recurrence of atrial fibrillation, the automatic optimization was set off at discharge. Consequently, the device did not optimize atrioventricular and interventricular delays (programming at discharge: 125 ms for the atrioventricular delay and 0 ms for the interventriculardelay). Our patient was treated with an anti-arrhythmic drug. Five months after implantation, his clinical status remained impaired (left ventricular ejection fraction = 30 %). The SonR signal amplitude had also decreased from 0.52 g to 0.29 g. Nevertheless, because our patient was no longer presenting with atrial fibrillation, the anti-arrhythmic treatment was stopped and the SonR optimization system was activated. After 2 months of automatic cardiac resynchronization therapy with defibrillator optimization, our patient’s clinical status had significantly improved (left ventricular ejection fraction = 60 %, New York Heart Association class II) and the SonR signal amplitude had doubled shortly after the first weekly automatic optimization.

**Conclusion:**

In this non-responding patient, device-based automatic cardiac resynchronization therapy optimization was shown to significantly improve his clinical status.

## Introduction

Cardiac resynchronization therapy (CRT) is an established therapy for patients with heart failure symptoms, left ventricular (LV) systolic dysfunction, and a wide QRS, on top of optimal medical therapy [1, 2]. However, the magnitude of clinical and hemodynamic benefit of CRT among recipients varies and non-responders can account for up to 30 % of treated patients [1]. The non-response can be partly caused by inappropriate settings of atrioventricular (AV) and interventricular (VV) delays leading to persistent AV, VV, and intraventricular dysynchrony. Several methods have been developed to optimize AV and VV delays, including device-based algorithms allowing automatic optimization of delays [3]. Encapsulated in the SonRtip atrial lead (Sorin CRM SAS, Clamart, France), the hemodynamic SonR sensor automatically optimizes AV and VV delays weekly in patients with heart failure in sinus rhythm, at rest, and during exercise [4].

The SonR sensor was clinically evaluated in the CLEAR multicenter, single-blind, randomized (1:1) pilot study (n = 199) using the SonR system integrated in a CRT device with a pacemaker. The primary effectiveness outcome was the response rate based on a hierarchical clinical composite score including (a) death, (b) heart failure-related hospitalization, (c) New-York Heart Association (NYHA) functional classification, and (d) quality of life using the EQ-5D questionnaire. Responders’ rate were 76 % in the SonR arm versus 62 % in the control arm (*p* = 0.03) at 1 year; this result was driven by improvement of symptoms [5]. A post-hoc analysis of the study showed that systematic optimization (n = 66, three times during one year, whether the method used SonR or echo) was associated with more responders as per the clinical composite score, fewer deaths or heart failure hospitalizations, and fewer symptoms versus non-systematic optimization (n = 133, 48 % of patients never optimized, 29 % optimized once, 23 % optimized twice), suggesting that favorable outcomes were associated with optimization frequency, not with the optimization method used [6]. A recent cost-effectiveness analysis found that this repeated optimization strategy was more cost-effective than the non-systematic optimization arm in most European countries [7]. Finally, Duncker *at al*. published a prospective, multicenter, non-randomized study designed to assess the safety and electrical performances of the atrial SonRtip lead in 99 patients implanted with a CRT device with a defibrillator (CRT-D) [8].

We describe a case report of a non-responding patient implanted with this atrial lead, who then responded to CRT after activation of the sensor.

## Case presentation

A 78-year-old Caucasian man presented with dilated ischemic cardiomyopathy, left bundle branch block, a left ventricular ejection fraction (LVEF) of 35 %, NYHA III/IV heart failure, diabetes, paroxysmal atrial fibrillation (AF), dyspnea when undergoing mild exercise, and edema of his lower limbs.

In December 2012, our patient was implanted with a triple chamber CRT-D device (Paradym RF SonR CRT 9770, Sorin CRM SAS, Clamart, France) and the atrial lead positioned in the lateral wall (560 Ω, 4.5 mV, 0.50 V at 0.35 ms). The right ventricular lead was a single coil implanted in the septum (659 Ω, 15.2 mV, 0.75 V at 0.35 ms) and a bipolar LV lead was inserted through the posterior vein (955 Ω, 0.75 V at 0.35 ms). Because of a recurrence of AF, the automatic optimization was set off at discharge. Consequently, the device recorded the hemodynamic SonR signal, but did not optimize AV and VV delays. Nominal AV and VV delays were programmed at hospital discharge (125 ms for AV and 0 ms for VV). Our patient’s anti-arrhythmic treatment consisted of amiodarone*,* 200 mg daily. While his QRS width was 195 ms before implant; it decreased down to 120 ms just after implantation. Echocardiography also showed a left ventricular end diastolic volume (LVEDV) of 135 mL and left ventricular end systolic volume (LVESV) of 85 mL.

**Fig. 1:**
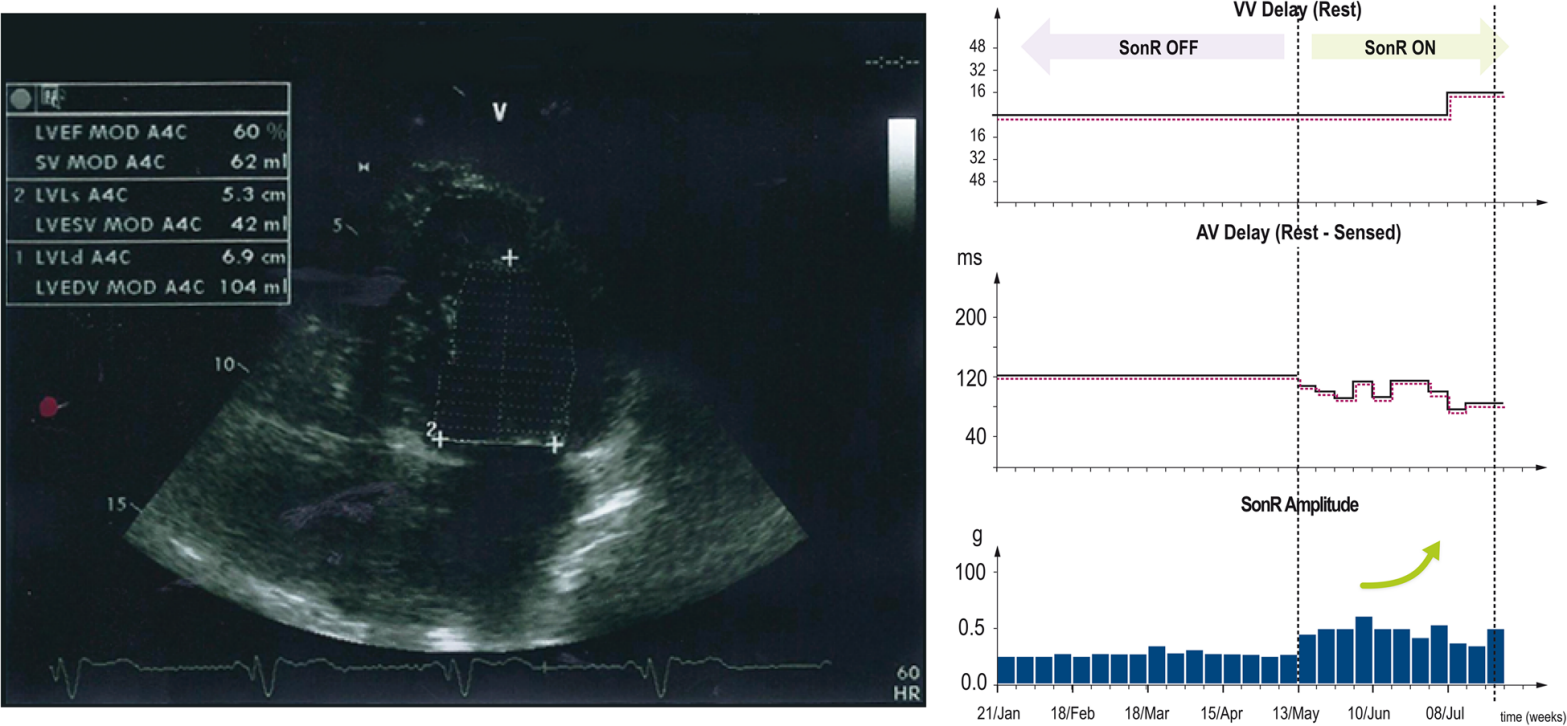
SonR parameters (interventricular delay at rest, sensed atrioventricular delay at rest, and SonR signal amplitude) before and after activation of the automatic optimization (in May 2013) and echocardiography

Five months after implant (in May 2013), our patient’s clinical status remained impaired, with a LVEF of 30 %, NYHA III/IV, QRS width of 96 ms, slight mitral regurgitation, LVEDV of 134 mL, and LVESV of 93 mL. The SonR signal amplitude had also decreased from 0.52 g to 0.29 g (Fig. 1). Nevertheless, because our patient no longer presented with AF (only one 6-day mode switch episode recorded shortly after implant), the anti-arrhythmic treatment was stopped and the SonR optimization system was activated.

After 2 months of automatic CRT-D optimization (7 months after implantation), our patient’s clinical status had significantly improved (LVEF of 60 %, NYHA II, no mitral regurgitation, optimal ventricular filing [E/A timing] with AV optimization, stable QRS width, LVEDV of 104 mL, and LVESV of 42 mL). His symptoms (dyspnea and lower limb edema) had disappeared at the 7-month post-implant visit. Optimized by the device, AV and VV delays at 85 ms and R+L 16 ms, respectively, were confirmed to be optimal both by echo and EA filling time. In addition, the SonR signal amplitude doubled shortly after the first weekly automatic optimization (Fig. 1).

The different echocardiographies of our patient were performed by the same operator.

## Discussion

CRT optimization could not initially be performed in our patient because of paroxysmal AF. Once the AF ceased, weekly AV and VV delay optimizations were automatically activated using a hemodynamic device-based sensor. Symptoms and ventricular function (LVEF, mitral regurgitation, ventricular filing) were significantly improved after 2 months of CRT optimization.

Significant clinical improvements after AV and VV optimization have previously been reported in two studies evaluating device-based optimization algorithms [5, 9]. The multicenter, single-blind, randomized (1:1) CLEAR trial compared CRT-P optimized using SonR and CRT optimized according to the centers’ usual practices, mostly by echocardiography. One-year results showed an improvement of symptoms (NYHA functional class) in 83 % of patients versus in 64 % of patients treated with CRT alone (*p* = 0.002). The evolution of QRS duration, LVESD, and LVEF was similar in both arms from baseline to 1 year [5].

In a recent paper by Oliveira *et al.*, 17 patients implanted with the SonRtip atrial lead and CRT-D device showed a significant increase in LVEF, with a 76.5 % rate of reverse remodeling, defined as an improvement of at least one NYHA functional class and a decrease >15 % of their LVESV at 6 months compared with baseline [10].

These pilot studies and preliminary results warrant an evaluation of the device in a controlled randomized trial. The double-blinded, multicenter, non-inferiority RESPOND- CRT trial will assess the clinical effectiveness and reverse remodeling of systematic automatic optimization versus a single echocardiographic optimization after implantation [11].

In this case report, QRS did not appear to be an index for CRT response, because it remained stable throughout the follow-up.

## Conclusions

Device-based automatic AV and VV delay CRT optimization significantly improved symptoms and ventricular function in a non-responding patient after 2 months.

## Consent

Written informed consent was obtained from the patient for publication of this case report and accompanying images. A copy of the written consent is available for review by the Editor-in-Chief of this journal.

## Abbreviations

AF: atrial fibrillation
AV: atrioventricular
CRT: cardiac resynchronization therapy
CRT-D: cardiac resynchronization therapy with a defibrillator
LV: left ventricular
LVEDV: left ventricular end diastolic volume
LVEF: left ventricular ejection fraction
LVESV: left ventricular end systolic volume
NYHA: New York Heart Association
VV: interventricular
